# Technical Framework for a Care Robot

**DOI:** 10.1093/geroni/igaf122.1545

**Published:** 2025-12-31

**Authors:** Momotaz Begum, Ola Ghattas, Marzan Alam, Moniruzzaman Akash, Noushad Sojib, Mostafa Hussein

**Affiliations:** University of New Hampshire, Durham, New Hampshire, United States; University of New Hampshire, Durham, New Hampshire, United States; University of New Hampshire, Durham, New Hampshire, United States; University of New Hampshire, Durham, New Hampshire, United States; University of New Hampshire, Durham, New Hampshire, United States; University of New Hampshire, Durham, New Hampshire, United States

## Abstract

A robot that cares for older adults in their home is tasked with making reliable sequential decisions subjected to uncertainties of human behaviors and environments. This is highly non-trivial due to a plethora of challenges involved with real-time processing of perception-action pipeline. The state of the art in AI robotics offers efficient tools to solve individual problems that may arise along this pipeline. An integrated solution, however, is still non-existent. We will present our ongoing work on developing an open-source control architecture that implements the entire perception-action pipeline for home care robots in a platform agnostic manner. The core of this architecture is a lightweight ROS2-based AI planner that can generate task plans executable by any robot – equipped with appropriate actuators – while sensing the current state of the environment and the care recipient (ROS: Robot Operating System; AI: Artificial Intelligence). Tasks typically included care services – e.g., reminding an older adult to take the medication or encouraging him/her to perform physical exercise – defined based on the needs of a care recipient. We integrate state of the art algorithms in robotics and computer vision – such as navigation, tracking, face recognition, natural language processing, etc. – in an innovative way with this lightweight AI planner to realize real-time planning and execution of a wide range of services that are critical for older adults to age gracefully in their own homes. We will report some data from our ongoing field studies.

